# Bone to pick: the importance of evaluating reference genes for RT-qPCR quantification of gene expression in craniosynostosis and bone-related tissues and cells

**DOI:** 10.1186/1756-0500-5-222

**Published:** 2012-05-08

**Authors:** Xianxian Yang, Jodie T Hatfield, Susan J Hinze, Xiongzheng Mu, Peter J Anderson, Barry C Powell

**Affiliations:** 1 Department of Plastic and Reconstructive Surgery, Shanghai Ninth People’s Hospital, Shanghai Jiao Tong University School of Medicine, Shanghai, China; 2 Women’s and Children’s Health Research Institute, Adelaide, Australia; 3 Australian Craniofacial Unit, Women’s and Children’s Hospital, Adelaide, Australia; 4 Discipline of Paediatrics, University of Adelaide, Adelaide, Australia

**Keywords:** Osteocalcin, Alkaline phosphatase, 18 S RNA, Gapdh, β-actin, geNorm, Normfinder, Craniosynostosis, Bone, Mineralization

## Abstract

**Background:**

RT-qPCR is a common tool for quantification of gene expression, but its accuracy is dependent on the choice and stability (steady state expression levels) of the reference gene/s used for normalization. To date, in the bone field, there have been few studies to determine the most stable reference genes and, usually, RT-qPCR data is normalised to non-validated reference genes, most commonly GAPDH, ACTB and 18 S rRNA. Here we draw attention to the potential deleterious impact of using classical reference genes to normalise expression data for bone studies without prior validation of their stability.

**Results:**

Using the geNorm and Normfinder programs, panels of mouse and human genes were assessed for their stability under three different experimental conditions: 1) disease progression of Crouzon syndrome (craniosynostosis) in a mouse model, 2) proliferative culture of cranial suture cells isolated from craniosynostosis patients and 3) osteogenesis of a mouse bone marrow stromal cell line. We demonstrate that classical reference genes are not always the most ‘stable’ genes and that gene ‘stability’ is highly dependent on experimental conditions. Selected stable genes, individually or in combination, were then used to normalise osteocalcin and alkaline phosphatase gene expression data during cranial suture fusion in the craniosynostosis mouse model and strategies compared. Strikingly, the expression trends of alkaline phosphatase and osteocalcin varied significantly when normalised to the least stable, the most stable or the three most stable genes.

**Conclusion:**

To minimise errors in evaluating gene expression levels, analysis of a reference panel and subsequent normalization to several stable genes is strongly recommended over normalization to a single gene. In particular, we conclude that use of single, non-validated “housekeeping” genes such as *GAPDH*, *ACTB* and *18 S rRNA*, currently a widespread practice by researchers in the bone field, is likely to produce data of questionable reliability when changes are 2 fold or less, and such data should be interpreted with due caution.

## Background

To help elucidate the molecular mechanisms involved in skull development and bone growth and the dysregulation that occurs during craniosynostosis, a medical condition that affects skull formation in 1 in 2500 live births, the ability to accurately measure changes in gene expression levels is vital. The most popular method is that of RT-qPCR where gene expression is measured against a reference gene. Alternatively, and less often, “absolute” quantification is used where gene expression is compared to an external standard and expressed relative to a biological unit, such as input RNA, cell number, or even a reference gene [1]. No studies have been published on the selection of suitable reference genes for use in craniosynostosis and most gene expression profiling on bone-related conditions rely on using common normalizers such as *ACTBGAPDH* and *18 S rRNA*, with little evidence of validation of their stability. Furthermore, most bone studies normalize data to only one reference gene, typically GAPDH (see Additional file 1), considered by many as a stable housekeeping gene despite substantial evidence to the contrary [1].

There is an increasing call to assess reference genes for stability in sample sets for each new experiment to avoid producing misleading data, particularly where the magnitudes of the gene expression changes are small. To address these concerns, a set of guidelines have been proposed; MIQE, minimum information for publication of quantitative real-time PCR experiments [2]. These guidelines invite a transparency in reporting so that the quality of data can be judged on several criteria such as experimental design, RNA quality control, normalization strategy and validation, data analysis and applied statistics. Unfortunately, recent studies published in the bone field indicate a widespread practice of reliance on normalising gene expression to non-validated, single gene references (see Additional file 1) and are indicative that there is insufficient awareness and appreciation of associated pitfalls.

In this study we investigated three common biological tools for studying osteogenesis and craniosynostosis in the laboratory; primary human cranial suture cells from affected patients, a mouse model of craniosynostosis and a mouse cell line to model osteogenesis and mineralisation in culture. Twelve candidate reference genes for human and mouse samples were chosen and assessed for stability using geNorm and Normfinder software. geNorm software uses geometric averaging of expression of a defined number of genes in a given cDNA sample set and determines the rank order of their relative stability [3] whereas Normfinder takes a “model-based approach” where input data is organised into groups (e.g. wildtype vs. mutant) and gene expression stability estimates take into account inter- and intragroup variations [4]. We compared the effect of normalizing data based on the least stable, most stable, and multiple stable reference genes for two common markers of osteogenesis, alkaline phosphatase (ALP) and osteocalcin (OC). Our results indicate substantial variability in the commonly used single housekeeping genes, *GAPDH, ACTB* and *18sRNA* across three experimental bone models and highlight the importance of validating and choosing the most appropriate combination of reference genes for each experimental dataset to avoid erroneous reporting of changes in gene expression levels in studies of bone biology.

## Results

### Selection of stable reference genes

Our panel of reference genes included members from distinct cellular pathways (i.e. less likely to be co-regulated) as well as classical housekeeping genes. RNA panels were selected to represent typical experiments in a bone lab: 1) mouse cranial suture tissues from *Fgfr2c*^*C342Y/+*^ mice harboring a Cys342Tyr replacement frequently observed in human Crouzon and Pfeiffer-type craniosynostosis, 2) cultured primary human cranial suture cells from craniosynostosis patients, and 3) a mouse osteoblastic cell line induced to mineralize over 21 days in culture (Table 1). Mineralisation was verified by the accumulation of Alizarin red S in induced samples relative to uninduced samples (Additional file 2). RNAs representative of each panel were chosen for geNorm and Normfinder analysis.

**Table 1:** RNA sample list

**Mouse suture tissues**						
**Time point**	**Genotype**	**Suture type**				
E16.5 *	Wildtype	Coronal, Posterior-frontal, Lambdoid, Parietal bone				
D0 ^#^	Wildtype	Coronal, Lambdoid				
D0	*Fgfr2c*^***C342Y/+***^	Posterior frontal, Sagittal				
D1	*Fgfr2c*^***C342Y/+***^	Coronal, Lambdoid				
D5	Wildtype	Lambdoid, Parietal bone				
D5	*Fgfr2c*^***C342Y/+***^	Posterior frontal, Sagittal				
D10	Wildtype	Coronal, Lambdoid				
D10	*Fgfr2c*^***C342Y/+***^	Posterior-frontal, Sagittal				
**Human suture cells**						
**Patient code**	**Phenotype**	**Sex**	**Age (months)**	**Suture type**	**Passage**	
AC 125	Sagittal synostosis	M	7	Unfused Coronal	P5, P10	
AC 124	Metopic synostosis	M	7	Unfused Coronal	P4, P8	
AC 126	Metopic synostosis	F	9	Unfused Coronal	P4	
AC 141	Sagittal synostosis	M	6	Unfused Coronal	P3	
AC 125	Sagittal synostosis	M	7	Unfused Lambdoid	P4	
AC 120	Crouzonoid syndrome	F	96	Fused Sagittal	P9	
AC 113	Multi-suture synostosis	F	10	Fused Coronal	P5	
AC 34	Multi-suture synostosis	F	5	Fusing Sagittal	P5	
**Kusa 4b10 - Osteogenesis assay**^**+**^						
**Induced cells**			**Uninduced cells**			
-			0			
3			3			
7			7			
14			14			
21			21			

*E refers to embryonic day.

^#^D refers to postnatal day.

^+^days after induction or days after the equivalent time point for uninduced cells.

### RT-qPCR analysis

RT-qPCR data was analyzed using geNorm software to obtain a stability value (M) for each reference gene and the mean pairwise variation value (V) in a sample set. Genes with the lowest M values were considered the most stable, while the V value indicated the optimal number of genes to use for normalization. The same data was then analysed with Normfinder and the two approaches compared. Stabilities of reference genes in our sample panels are shown in Additional files 3 and 4 and summarized in Table 2.

**Table 2:** Summary of geNorm and Normfinder gene stability values

**Mouse suture tissues**				**Human suture cells**				**Kusa 4b 10 cells**			
**geNorm**		**Normfinder**		**geNorm**		**Normfinder**		**geNorm**		**Normfinder**	
18 S rRNA	0.95	Eif4a2	0.477	ACTB	0.77	ACTB	0.716	Rpl13a	0.67	Gapdh	0.249
Eif4a2	0.84	18 S rRNA	0.390	EIF4A2	0.67	EIF4A2	0.687	Gapdh	0.62	Rpl13a	0.237
Sdha	0.75	Atp5b	0.340	SDHA	0.63	GAPDH	0.465	B2m	0.56	B2m	0.200
Rpl13a	0.67	Sdha	0.315	B2M	0.60	SDHA	0.461	Sdha	0.50	Sdha	0.184
B2m	0.63	Cyc1	0.289	TOP1	0.57	TOP1	0.422	Ywhaz	0.48	Ywhaz	0.135
Actb	0.59	Ywhaz	0.274	RPL13A	0.53	B2M	0.348	Ubc	0.44	Canx	0.097
Atp5b	0.55	Actb	0.255	SF3A1	0.49	CYC1	0.337	Actb	0.42	Ubc	0.091
Ywhaz	0.52	Gapdh	0.224	YWHAZ	0.41	YWHAZ	0.337	Atp5b	0.39	Actb	0.086
Ubc	0.49	Rpl13a	0.194	CYC1	0.36	ATP5B	0.295	Canx*	0.35	**Atp5b**	0.074
**Canx**	0.47	B2m	0.188	GAPDH	0.31	**18 S rRNA**	0.262	18 s rRNA*	0.35	**18 S rRNA**	0.048
Cyc1*	0.34	**Ubc**	0.141	18 S rRNA*	0.28	RPL13A	0.217				
Gapdh*	0.34	**Canx**	0.081	ATP5B*	0.28	**SF3A1**	0.202				

Genes are ranked from the least stable (top) to the most stable (bottom). Stability values are shown for geNorm and Normfinder and lower numbers indicate greater stability. Asterisk indicates equal ranking. The optimal number of reference genes to use, based on their V values (geNorm) or intergroup/intragroup variations (Normfinder) are marked as bold.

In our first test panel we determined the most stable reference genes in craniosynostosis-related suture material from a commonly used mouse model for Crouzon syndrome. The geNorm rank order data analysis indicated that *Cyc1, Gapdh* and *Canx* were the most stable combination of reference genes to use, while *18 S rRNA* gene had the highest variability (Table 2; Additional file 3). Normfinder also ranked *18 s rRNA* as one of the least stable genes and *Canx* as one of the most stable, with *Cyc1* and *Gapdh* ranked towards the middle (Table 2; Additional file 4). It proposes the use of *Ubc* and *Canx* as the most stable normalisation factor, and we note that *Ubc* is also considered an adequately stable gene by geNorm ranking (ranked below the M = 0.5 cutoff proposed by Vandesompele et al (2002).

We next determined if stability differed when switching to a different but related sample background, as it is a common laboratory habit to use the same “housekeeping” gene for all purposes, regardless of species, tissue source or process. Our second panel consisted of human cells sourced from the cranial sutures of craniosynostosis patients that have been subsequently cultured *in vitro*. Again geNorm ranked *CYC1* and *GAPDH* among the most stable genes, but they were superseded by *18 S rRNA* and *ATP5B*, which were considered sufficient for reliable normalization (Table 2; Additional file 3). Normfinder also ranked *18 S rRNA* and *ATP5B* among the most stable genes but recommended a combination of *18 S RNA* and *SF3A1* for normalization (Table 2; Additional file 4). It was striking that *18 S rRNA* was the most stable gene in the human cells whereas in mouse tissue it was the least stable. Significantly, another commonly used reference gene, *ACTB*, was ranked the least stable by both geNorm and Normfinder in the human cranial cells. We also noted that *GAPDH* was ranked very differently by the different software (geNorm – more stable, Normfinder – less stable) and assume this is a result of the different approaches each program takes.

In our final test panel we looked at the stability of our reference genes during terminal cell differentiation by following osteogenesis of the Kusa 4b 10 cell line over 21 days in culture. Genes *Cyc1* and *Eif4a2* were excluded from this analysis because of low abundance indicated by poor amplification. The two most stable genes, as determined by geNorm, were *Canx* and *18 S rRNA*, which were ranked as sufficient for reliable normalization among the remaining 10 candidate genes (Table 2; Additional file 3). *Atcb* was amongst the more stable genes while *Gapdh* was amongst the least stable. Normfinder rankings in this case are almost identical to that of geNorm, ranking *18 S rRNA* and one of the most stable and *Gapdh* as the least stable of the 10 genes (Table 2; Additional file 4). It recommends a normalization factor based on *18 S rRNA* and *Atp5b*.

### Expression patterns of two common markers of osteogenesis were significantly affected by the normalization strategy

To put the ranking analysis data into an experimental context we investigated the effect of reference gene selection on the expression profiles of two commonly used marker genes for bone development and mineralisation, OC and ALP. OC is a marker of terminally differentiated osteoblasts during osteogenesis and osteoblastogenesis [5,6], while ALP is a marker of early stage osteoblast differentiation [7,8]. Using RNA from cranial sutures of *Fgfr2c*^*C342Y/+*^ mice and their wildtype littermates, we expected to see an increase in OC and ALP expression during post-natal suture development [9,10]. Furthermore, as coronal sutures remain patent in wildtype mice and undergo premature bony fusion in *Fgfr2c*^*C342Y/+*^ mice, we also expected these genes to be differentially expressed between the two groups.

**Figure 1:**
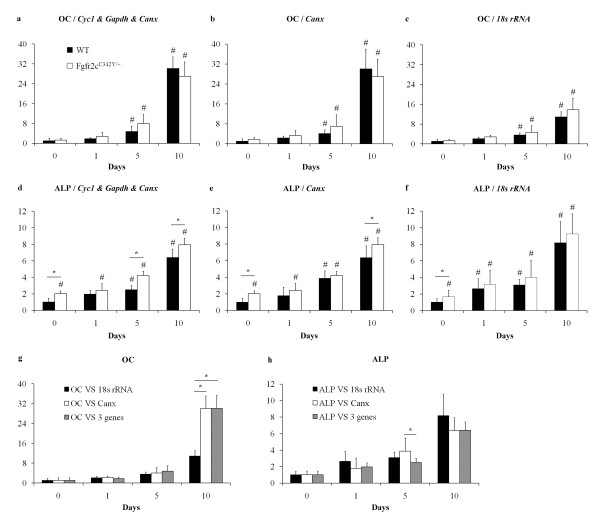
**Comparison of normalization strategies on expression of two common osteogenesis markers.** Relative quantification of OC and ALP expression during suture fusion in *Fgfr2c*^*C342Y/+*^ mice is dependent on which reference genes are used. Target gene expression was normalized to the geometric mean of *Cyc1* - *Gapdh* - *Canx* (**a** and **d**), the single most stable gene - *Canx* (**b** and **e**) or the least stable gene – *18 S rRNA* (**c** and **f**). Relative gene expression in wildtype sutures, normalized to the different reference genes, is also presented (g and h). Expression is displayed as fold change relative to day 0 wildtype. N = 6. * Represents a significant difference between two groups ( p < 0.05). # Represents a significant difference to Day 0 WT value (p < 0.05).

From the geNorm analysis of the mouse suture panel we applied *Canx* (one of the most stable genes), *18 S rRNA* (least stable gene) or *Cyc1-Gapdh-Canx* (recommended gene combination for normalization) as references for relative quantification of OC and ALP during suture fusion of *Fgfr2c*^*C342Y/+*^ mice and their wildtype littermates (Figure 1). We chose the geNorm rankings over the Normfinder rankings as geNorm was conveniently integrated into the qBasePLUS analysis software that was used to handle the many calculations required [11]. However, we note that Normfinder also ranks *Canx* as the most stable gene and *18 S rRNA* as second least stable gene.

Using any of the three normalizers, OC expression increased with age and showed no significant differences between the wildtype and mutant mice (Figure 1a-c). However, the use of *18 S rRNA* as the normalizer (least stable) resulted in a day 10 expression level that was approximately one third of that calculated by the other two strategies (Figure 1g).

Results for ALP expression also indicated a general increase in expression levels with age for all three normalisation strategies (Figure 1d-f). Using the three gene reference set, the results indicated that coronal sutures from *Fgfr2c*^*C342Y/+*^ mice expressed significantly more ALP than their wildtype counterparts for three of the four time points investigated. In contrast, ALP gene expression data generated using the *18 S rRNA* reference (the least stable), identified only Day 0 samples as significantly different and analysis based on the *Canx* reference (the most stable) indicated two significant differences. This outcome most likely reflects the small fold changes between the mutant and wildtype data points. We conclude that when judging the significance of values separated by a two fold change or less, small fluctuations in the data caused by the choice of normalization strategy can lead to significantly different outcomes and interpretations (Figure 1h).

Based on the use of the recommended normalisation strategy, our data indicates that *Fgfr2c*^*C342Y/+*^ mice have slightly elevated levels of ALP compared to their wildtype counterparts. Interestingly, although this suggests a possible increase in the number or activity of cells committing to the osteogenic cell lineage, there is no significant change in levels of the terminal differentiation marker OC between mutant and wildtype. Biological significance aside, it is clear to see that the normalization strategy has a strong influence over the magnitude (see OC) and number of significantly different data points (see ALP). Had we relied on *18 S rRNA* as the reference gene, we would have reported only a 10 fold increase in OC expression by day 10 (instead of ~30) but, more importantly, the significant differences in ALP expression between wildtype and mutant would not have been detected.

## Discussion

Traditionally, RT-qPCR has relied on normalization of gene expression levels to only one of a few housekeeping genes which were originally, and incorrectly, thought to be universally expressed at a steady-state level eg. *ACTB, 18 S rRNA* and *GAPDH*. Recently, this trend has begun to slowly change due to reports of the need to carefully select validated reference genes to avoid generating biased data due to variation in the reference gene’s expression pattern [2,3], [11]. However, our brief survey of the bone field indicates the practice of using a single, non-validated reference gene, typically *GAPDH* (followed by either *ACTB* or *18 S rRNA*), is still widespread. Here, we demonstrated that reference genes for normalizing data related to bone growth and craniosynostosis studies cannot be used interchangeably for different experimental situations, even if the samples are biologically similar. Notably, we found that the expression of *GAPDH, ACTB* and *18 S rRNA* genes changed the most between sample panels, indicating that of all the genes examined they were the least predictable in terms of stability. A recent geNorm-based study by Di et al (2011) confirms the variability of these three genes in other bone cell culture models although the authors did not investigate the potential adverse effects that might occur if normalization is restricted to one of those typical housekeeping genes [12].

Several studies show that when normalizing expression data, noticeable variations are produced by substituting different reference genes [13-17]. In mouse cranial suture tissue samples, we demonstrated the extent of this effect in a bone-specific context by using three normalization strategies to determine the expression levels of two common markers of osteogenesis and mineralization, OC and ALP. Expression of OC and ALP are regularly used as measures of osteogenesis and mineralisation and, as we have shown, use of even the single most stable reference gene can substantially bias data analysis, particularly when making judgements on the significance of low order magnitude changes (e.g. 2 fold). It is therefore of great concern that, based on our survey of the bone field, many recent studies of endochondral ossification have concluded significant changes in gene expression at a level of 2 fold or less based on the non-validated use of GAPDH, one of the most variable genes and amongst the least stable of genes in our cell culture studies in an endochondral ossification model.

Observation of the trends in any one of our three panels for OC or ALP expression in isolation would not necessarily indicate problematic data. Without prior knowledge of the ranking order provided, in this case by geNorm analysis, the observer would not be able to confidently select which strategy to use. Even selecting the single most stable reference gene, while a reasonable alternative when assessing OC expression, would be a poor choice for ALP expression.

There are currently three methods available for estimating reference gene expression stability based on experimental data, geNorm, Normfinder and Bestkeeper, and a fourth source, RefGenes, based on microarray data [3,4,18,19]. The two most popular (based on citation) are geNorm and Normfinder, which we compared in this study. In general, both programs identified the same genes as least stable and most stable although the individual ranking orders differed. This was not unexpected as the programs use two different approaches in order to rank gene expression stability. We can conclude that either program will generate a good starting point for selecting genes to use in normalization, but as we have shown here and as stated in the original articles for both geNorm [3] and Normfinder [4], generating a normalisation reference to multiple genes should always be considered.

Detection of gene expression changes during bone growth or disease progression can be a useful means of predicting outcomes for these processes. In theory, studies that can show significant up or down regulation of genes before the onset of gross morphological changes seen in craniosynostosis (fusing sutures) can help to identify important pivotal genes. Furthermore, the early detection of altered gene expression can help to pinpoint appropriate intervention times for applying new treatments. As shown in this study, the accuracy of this type of data is heavily dependent on choosing an appropriate normalisation strategy. The use of biased results, based on misleading trends or fold changes, could lead to misguided research and mistaken assessment of cause and effect.

## Conclusions

We strongly recommend that gene expression data be normalized to at least two validated reference genes. These could be determined by a geNorm-style selection process from a panel of candidate genes. In particular, we conclude that use of non-validated “housekeeping” genes such as *GAPDH*, *ACTB* and *18 S rRNA*, currently a widespread practice by researchers in the bone field, is likely to produce data of questionable reliability when changes are 2 fold or less, and such data should be considered with due caution.

## Methods

### Mouse suture tissues

Suture samples were collected from *Fgfr2c*^*C342Y/+*^ x Swiss mice offspring (E16.5, 0, 1, 5 and 10 day old mice). The posterior frontal, sagittal, coronal and lambdoid sutures were excised from the calvaria, leaving a thin strip of bone less then 1 mm along each side of the suture. Parietal bone was also collected. Pericranium and dura mater were dissected free and RNA extracted. Genotyping PCR on tail DNA (QIAGEN blood and tissue kit, Doncaster, Victoria, Australia) used primers 5’-CAAGCAAGCTCAACAGGAGAG-3’ and 5’-GCTGTGCTGCTGAGAGTTTTG-3’ producing a 224 bp wildtype amplicon or a 290 bp mutant amplicon. Adult C57Bl/6 skull, liver and brain and 3 week old femoral and tibial bones were processed for RNA. Work was approved by the Animal Ethics Committee, Children’s, Youth and Women’s Health Service, SA.

### Human suture cells

Human cranial suture cells were isolated from seven patients undergoing transcranial surgery for syndromic or non-syndromic craniosynostosis. Consent was obtained following the guidelines of the Research Ethics Committee of the Children, Youth and Women’s Health Service, SA. Patients were genotyped for mutations in *FGFR1-3* and *TWIST*[20]. Primary suture cells were obtained by collagenase digestion and explant culture [21]. Cells from different suture types and fusion states were used (Table 1). Cells were collected for RNA at various passages.

### Mouse Kusa 4b 10 cells

The Kusa 4b 10 bone marrow stromal cell line [22] was maintained in alpha MEM (Invitrogen, Sydney, Australia) supplemented with 10 % heat-inactivated fetal bovine serum (FBS, Invitrogen), 100 IU/ml penicillin and 100 IU/ml streptomycin (Sigma-Aldrich, Sydney, Australia). For osteogenesis, 5000 cells at P20 were plated in triplicate and induced the next day by addition of 10 mM β-glycerophosphate supplemented growth media (alpha MEM, 15 % FBS, 50 μg/mL ascorbic acid, 100 IU/ml penicillin and 100 IU/ml streptomycin) exchanged every three days for 21 days. Cells were either collected for RNA or fixed and stained with 0.1 % Alizarin red S solution (Sigma-Aldrich). Alizarin dye was destained and quantified at A_450_.

### RNA isolation and cDNA synthesis

Total RNA was extracted using Trizol (Invitrogen) with modifications for mouse suture and bone tissues as follows: tissues were homogenized in liquid nitrogen and RNA precipitation was aided by addition of glycogen. RNA pellets were reconstituted at 60°C for 10 min. For embryonic mouse suture samples, two mice of the same genotype were combined. For all samples, RNA quality was checked on a 1 % agarose gel and concentrations and A_260_/A_280_ ratios determined.

Total RNA (500 ng for mouse suture tissues, 250 ng for human suture cells and 1 μg for Kusa 4b 10 cells and other tissues) was reverse-transcribed using random hexamers (Superscript III first strand synthesis kit, Invitrogen). cDNA samples were diluted 1:3 in RNase/DNase free water before use in PCR; human suture cell cDNAs were diluted 1:4; Kusa 4b 10 cell cDNAs and adult tissues were diluted 1:10. No-RT controls were made for all mouse RNA samples by replacing Superscript III with RNase/DNase free water during the RT step. Representative no-RT controls were made for the human samples.

### geNorm/Normfinder Assays

geNorm assays were carried out using either the mouse or human geNorm^TM^ Housekeeping Gene Selection Kit (PrimerDesign Ltd, Southampton, UK). Genes are listed in Table 3. We chose this kit as the primer assays were guaranteed by the company to have high efficiencies, the gene group included members from distinct cellular pathways (i.e. less likely to be co-regulated) and they included a number of classical housekeeping genes which are in common use in the bone field and which were therefore important to assess. Kusa 4b 10 PCR assays used 5 μl cDNA, 0.12 μl 100×SYBR green (Thermo Fisher Scientific, Victoria, Australia), 2 μl 10×PCR Buffer (Applied Biosystems, Victoria, Australia), 2 μl 25 mM MgCl_2_ (Applied Biosystems), 0.2 μl 10 mM dNTPs (Invitrogen), 300 nM primers (PrimerDesign Ltd), 5 U/μl AmpliTaqGold DNA polymerase (Applied Biosystems) to a final volume of 20 μl. Cycling conditions were as follows: an initial 95°C step for 10 min, then 40 cycles of 95°C for 15 s and finally 60°C for 60 s. KAPA SYBR FAST qPCR Master Mix (Kapa Biosystems, Woburn, MA, USA) was used for mouse suture tissue and human suture cells assays. 10 μl qPCR reactions contained 5 μl 2×Kapa Master Mix, 0.5 μl gene specific 20×primer mix, 1.43 μl cDNA (diluted 1:3 or 1:4) and 3.07 μl RNase/DNase free water. The thermal profile was 95°C for 3 min followed by 40 cycles of 30 s at 95°C and 25 s at 60°C. All samples were assayed in duplicate, including no template controls (NTCs), on a Rotor-Gene 6000 Real-time PCR machine (Qiagen). Melt curve analysis, gel electrophoresis and sequencing were used to verify product identity. For stability comparisons of candidate reference genes, geNorm software version 3.5 and Normfinder was used [3,4].

**Table 3:** geNorm housekeeping gene selection kit candidates

**Gene Symbol**	**Gene Name**	**Pathway/Process**
**geNorm Housekeeping Gene Selection Kit - Mouse**		
*Actb*	β-actin	cytoskeletal protein
*B2m*	β-2 microglobin	MHC class 1 protein
*Gapdh*	Glyceraldehyde-3-phosphate dehydrogenase	glycolysis
*Ywhaz*	Phospholipase AZ	signal transduction
*Rpl13a*	Ribosomal protein L13A	protein synthesis
*Cyc1*	Cytochrome c-1	electron transport chain
*Sdha*	Succinate dehydrogenase complex subunit A	citric acid cycle
*18 S rRNA*	18 S ribosomal RNA	protein synthesis
*Eif4a2*	Eukaryotic translation initiation factor 4A2	protein synthesis/RNA helicase
*Atp5b*	ATP synthase subunit 5B	mitochondrial ATP synthesis
*Ubc*	Ubiquitin C	protein turnover
*Canx*	Calnexin	protein folding
**geNorm Housekeeping Gene Selection Kit - Human**		
*ACTB*	β-actin	cytoskeletal protein
*B2M*	β-2 microglobin	MHC class 1 protein
*GAPDH*	Glyceraldehyde-3-phosphate dehydrogenase	glycolysis
*YWHAZ*	Phospholipase AZ	signal transduction
*RPL13A*	Ribosomal protein L13A	protein synthesis
*CYC1*	Cytochrome c-1	electron transport chain
*SDHA*	Succinate dehydrogenase complex subunit A	citric acid cycle
*EIF4A2*	Eukaryotic translation initiation factor 4A2	protein synthesis
*18 S rRNA*	18 S ribosomal RNA	protein synthesis/RNA helicase
*ATP5B*	ATP synthase subunit 5B	mitochondrial ATP synthesis
*SF3A1*	Splicing factor 3 subunit 1	transcription
*TOP1*	DNA topoisomerase 1	DNA replication

### Evaluation of selected reference genes in a mouse craniosynostosis study

RT-qPCR was carried out on 48 mouse coronal suture tissue samples collected from 6 wild-type and 6 *Fgfr2c*^*C342Y/+*^ mice at days 0, 1, 5 and 10. Gene expression was measured for OC (Forward: 5’-ACCTCACAGATGCCAAGCC-3’, Reverse: 5’-ATCTGGGCTGGGGACTGAG-3’) and ALP (Forward: 5’-GGGACGAATCTCAGGGTACA-3’, Reverse: 5’-AGTAACTGGGGTCTCTCTCTTT-3’) and normalized to the most stable, the three most stable or the least stable reference gene(s) using qBasePLUS software (Biogazelle, Ghent, Belgium) [11]. Standard curves were obtained for each assay showing the qPCR reaction efficiency to be 100 ± 5 % and were included in calculations.

## Abbreviations

(RT-qPCR): Reverse transcription - quantitative PCR; (ALP): Alkaline phosphatase; (OC): Osteocalcin; (NTC): No template control.

## Competing interests

Funding was received from the NHMRC, WCH Research Foundation, Australian CranioMaxilloFacial Foundation, the Science and Technology Commission of Shanghai Municipality, project 10410701300, ‘Cellular and Animal Model Study in Craniosynostosis’and a Grant-in-aid from Denis Harwood. The authors have no financial interests in this research project or in any of the techniques or equipment used in this study. The authors declare that they have no competing interests.

## Authors' contributions

JTH conceived the study. JTH, XY, PJA and BCP wrote the manuscript. XY carried out the mouse suture tissue studies. JTH & SJH carried out the Kusa 4b 10 studies. SJH carried out the human suture cell studies. PJA collected human suture samples. JTH, XY, SJH, XM, PJA and BCP discussed the data. All authors read and approved the final manuscript.

## Authors' information

JTH, SJH and BCP are molecular biologists in the bone field. XY, XM and PJA are craniofacial surgeons. PJA and BCP are joint senior authors.

## Supplementary Material

Additional file 1 Simple and brief survey of the literature for articles that presented bone-related RT-qPCR data in 2011–2012. Click here for file

Additional file 2 Induction of mineralisation in Kusa 4b 10 cells. Click here for file

Additional file 3 Reference gene stability ranking in three bone-related experimental groups using geNorm analysis. Click here for file

Additional file 4 Reference gene stability ranking in three bone-related experimental groups using Normfinder analysis. Click here for file
