# Supplementary material for: Bone to pick: the importance of evaluating reference genes for RT-qPCR quantification of gene expression in craniosynostosis and bone-related tissues and cells
Source: BMC Res Notes. 2012 May 8;5:222. doi: 10.1186/1756-0500-5-222 (PMC3476976; doi:10.1186/1756-0500-5-222)
Supplement: Additional file 1 — Simple and brief survey of the literature for articles that presented bone-related RT-qPCR data in 2011–2012. [file 1756-0500-5-222-S1.pdf]

**Bone to pick: the importance of evaluating reference genes for RT-qPCR quantification of gene expression in craniosynostosis and bone-related tissues and cells**

Xianxian Yang, Jodie T Hatfield, Susan J Hinze, Xiongzheng Mu, Peter J Anderson and Barry C Powell

## Additional file 1.pdf

## Simple and brief survey of the literature for articles that presented bone-related RT-qPCR data in 2011-2012.

### Aim

We wished to determine how prevalent the use of validated reference genes was in current bone-related RT-qPCR studies, and also whether single or multiple reference genes were used.

### Method

Using the PubMed search engine, we limited the searches to the year 2011-2012 (year to date) and to articles that were open access and in English. The search was refined by using search terms:

Search A: osteogenesis AND gene AND expression AND PCR AND real-time
Search B: gene expression OR RT-PCR OR quantitative PCR - limited to the journal "Bone"

The two different searches were carried out to survey trends in bone-related literature in journals in general and in literature from a bone specific journal. We manually identified the reference gene/s used for normalization and noted if evidence of validation had been presented. This was done for the 15 most recent articles as defined by each search pattern and the findings are presented in table form.

### Results

Presented in Tables 1 and 2 on the following pages.

### Conclusions

1. 93% (23/30) of RT-qPCR studies surveyed used single, non-validated genes to normalize data.
2. The classical housekeeping gene *GAPDH* was the most commonly used (57%), followed by *18S rRNA* (13%) and *ACTB* (13%).

**Bone to pick: the importance of evaluating reference genes for RT-qPCR quantification of gene expression in craniosynostosis and bone-related tissues and cells**
Xianxian Yang, Jodie T Hatfield, Susan J Hinze, Xiongzheng Mu, Peter J Anderson and Barry C Powell

## Table 1

| | Search A | PMID: | Reference gene | Validation | Multiple reference genes |
|---|---|---|---|---|---|
| 1 | J Oral Sci. 2011 Dec;53(4):509-16 | 22167038 | GAPDH | no | no |
| 2 | Gene. 2012 Jan 10;491(2):187-93. Epub 2011 Oct 12 | 22019432 | GAPDH | no | no |
| 3 | Cell Prolif. 2011 Oct;44(5):420-7. | 21951285 | GAPDH | no | no |
| 4 | Clin Oral Investig. 2011 Sep 22 [Epub ahead of print] | 21938481 | GAPDH | no | no |
| 5 | Int J Biol Sci. 2011;7(7):968-77. Epub 2011 Aug 7 | 21850206 | 18S rRNA | no | no |
| 6 | Lasers Med Sci. 2011 Aug 13. [Epub ahead of print] | 21842225 | GAPDH | no | no |
| 7 | J Biol Chem. 2011 Sep 23;286(38):32995-3002. Epub 2011 Aug 2 | 21828043 | GAPDH | no | no |
| 8 | Biochem Biophys Res Commun. 2011 Aug 19'412(1):143-9. Epub 2011 Jul 22 | 21806974 | RPL13A | no | no |
| 9 | Biochem Biophys Res Commun. 2011 Aug 12;411(4):690-4. Epub 2011 Jul 13 | 21772583 | GAPDH | no | no |
| 10 | Eur Cell Mater. 2011 Jul 15;22:68-82; discussion 83 | 21761393 | GAPDH | no | no |
| 11 | Platelets. 2011;22(6):452-60. Epub 2011 Jul 11 | 21740339 | GAPDH | no | no |
| 12 | Biomaterials. 2011 Oct;32(29):6900-11/ Epub 2011 Jul 5 | 21733571 | GAPDH | no | no |
| 13 | Mol Cell Biochem. 2011 Dec;358(1-2):37-44. Epub 2011 Jun 23 | 21698346 | GAPDH | no | no |
| 14 | Biotechnol Lett. 2011 Oct;33(10):1933-8. Epub 2011 Jun 10 | 21660578 | ACTB | no | no |
| 15 | Bone. 2011 Sep;49(3):463-72. Epub 2011 Apr 27. | 21549232 | GAPDH | no | no |

**Bone to pick: the importance of evaluating reference genes for RT-qPCR quantification of gene expression in craniosynostosis and bone-related tissues and cells**

Xianxian Yang, Jodie T Hatfield, Susan J Hinze, Xiongzheng Mu, Peter J Anderson and Barry C Powell

## Table 2

| | Search B | PMID: | Reference gene | Validation | Multiple reference genes |
|---|---|---|---|---|---|
| 1 | Bone. 2012 Feb;50(2):467-76. Epub 2011 Oct 14. | 22019459 | GAPDH | no | no |
| 2 | Bone. 2012 Feb;50(2):499-509. Epub 2011 Aug 2. | 21839191 | L24/ACTB/cyclophilin | no | yes |
| 3 | Bone. 2012 Feb;50(2):546-52. Epub 2011 Jun 24 | 21723971 | 18S rRNA or FABP4/aP2 | no | no |
| 4 | Bone. 2012 Jan 21. [Epub ahead of print] | 22285620 | ACTB | no | no |
| 5 | Bone. 2012 Jan 16. [Epub ahead of print] | 22270057 | GAPDH | no | no |
| 6 | Bone. 2012 Jan;50(1):364-72. Epub 2011 Nov 12. | 22108137 | ACTB | no | no |
| 7 | Bone. 2012 Jan;50(1):337-42. Epub 2011 Nov 10. | 22100507 | YWHAZ, UBC and TOP1 | yes | yes |
| 8 | Bone. 2012 Jan;50(1):265-75. Epub 2011 Nov 4. | 22079283 | ACTB | yes | no |
| 9 | Bone. 2012 Jan;50(1):209-17. Epub 2011 Oct 30. | 22075208 | ChoB | no | no |
| 10 | Bone. 2012 Jan;50(1):171-81. Epub 2011 Oct 18. | 22037351 | U6 snRNA or GAPDH | no | no |
| 11 | Bone. 2012 Feb;50(2):467-76. Epub 2011 Oct 14. | 22019459 | GAPDH | no | no |
| 12 | Bone. 2011 Dec;49(6):1311-6. Epub 2011 Sep 22. | 21964323 | GAPDH | no | no |
| 13 | Bone. 2012 Jan;50(1):54-62. Epub 2011 Sep 16. | 21964322 | 18s rRNA | no | no |
| 14 | Bone. 2012 Jan;50(1):79-84. Epub 2011 Sep 10. | 21930256 | 18s rRNA | no | no |
| 15 | Bone. 2012 Jan;50(1):28-41. Epub 2011 Aug 31. | 21911092 | HPRT1 | no | no |
