# Supplementary material for: Bone to pick: the importance of evaluating reference genes for RT-qPCR quantification of gene expression in craniosynostosis and bone-related tissues and cells
Source: BMC Res Notes. 2012 May 8;5:222. doi: 10.1186/1756-0500-5-222 (PMC3476976; doi:10.1186/1756-0500-5-222)
Supplement: Additional file 2 — Induction of mineralisation in Kusa 4b 10 cells. [file 1756-0500-5-222-S2.pdf]

Bone to pick: the importance of evaluating reference genes for RT-qPCR quantification of gene expression in craniosynostosis and bone-related tissues and cells

Xianxian Yang, Jodie T Hatfield, Susan J Hinze, Xiongzheng Mu, Peter J Anderson and Barry C Powell

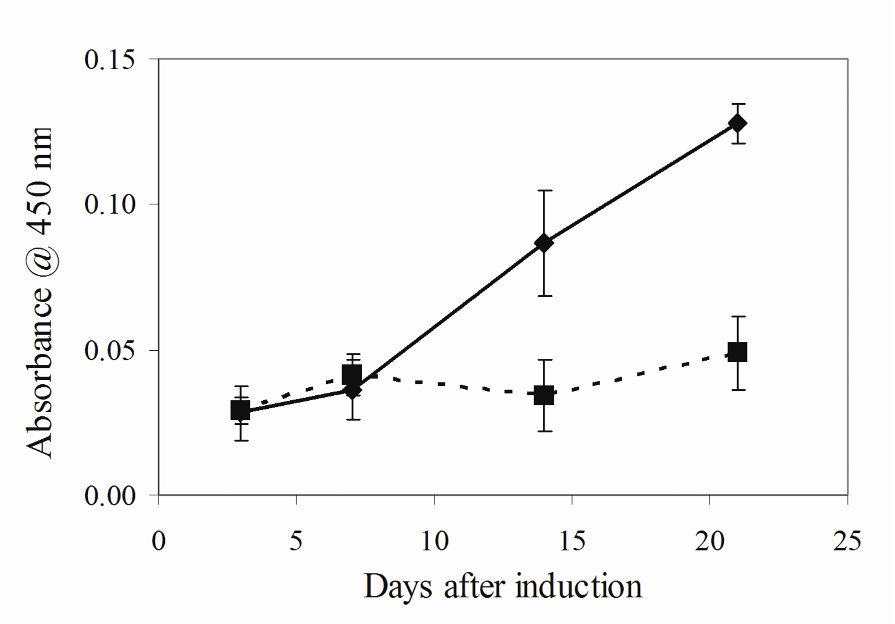

Additional file 2

Induction of mineralisation in Kusa 4b 10 cells

Cells were either uninduced (dotted line) or induced to differentiate into osteoblasts by addition of ß–glycerophosphate (solid line). The progression of mineralisation over 21 days was measured by an increase in absorbance (A_{450}) of Alizarin red S extracted from fixed and stained cells. After two weeks, induced cells at days 14 and 21 showed a significant increase in mineralisation compared to day 0 or uninduced cells (*$P<0.05$, Student t-test). N=3. Average ± SD shown.
