# Supplementary material for: Bone to pick: the importance of evaluating reference genes for RT-qPCR quantification of gene expression in craniosynostosis and bone-related tissues and cells
Source: BMC Res Notes. 2012 May 8;5:222. doi: 10.1186/1756-0500-5-222 (PMC3476976; doi:10.1186/1756-0500-5-222)
Supplement: Additional file 3 — Reference gene stability ranking in three bone-related experimental groups using geNorm analysis. [file 1756-0500-5-222-S3.pdf]

Bone to pick: the importance of evaluating reference genes for RT-qPCR quantification of gene expression in craniosynostosis and bone-related tissues and cells

Xianxian Yang, Jodie T Hatfield, Susan J Hinze, Xiongzheng Mu, Peter J Anderson and Barry C Powell

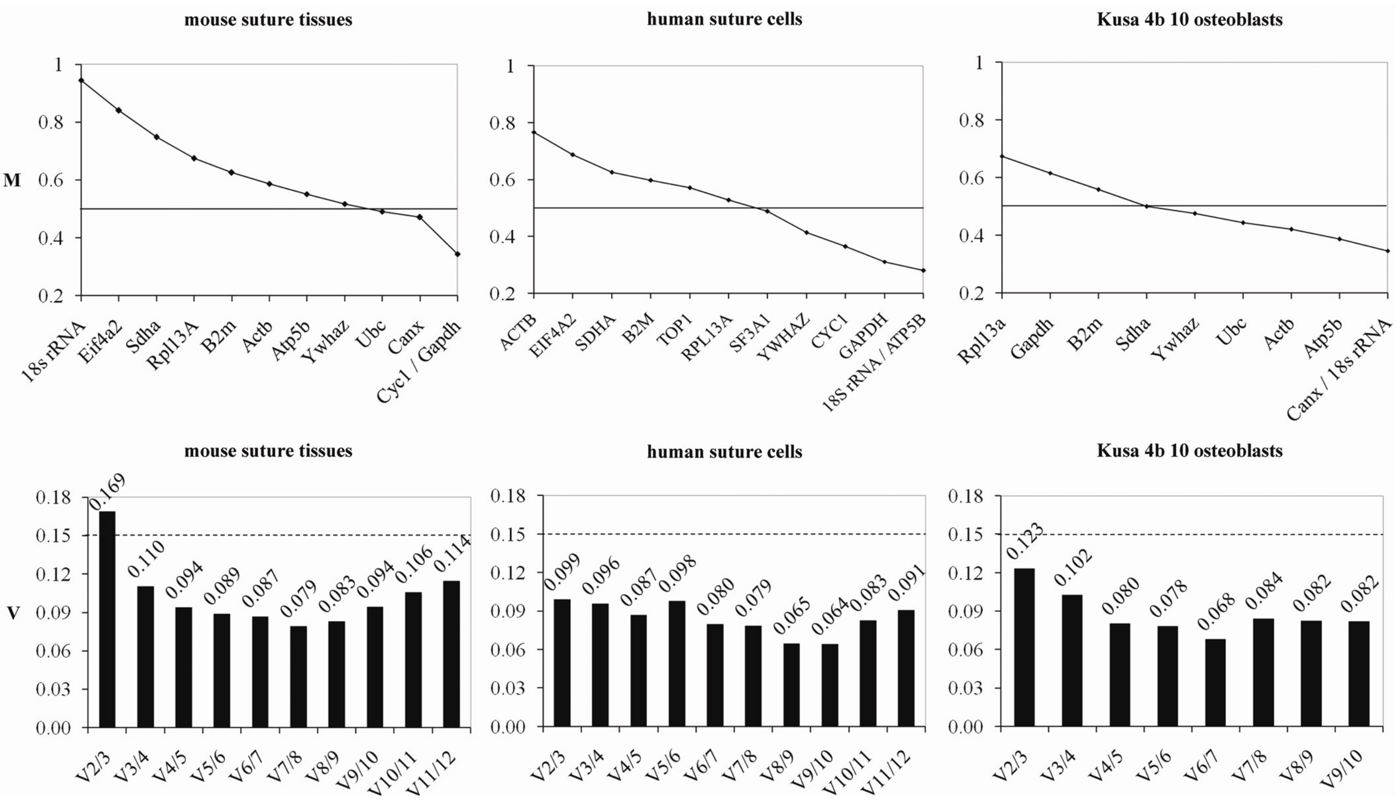

Additional file 3

Reference gene stability ranking in three bone-related experimental groups using geNorm analysis

GeNorm analyses were carried out on craniosynostosis-related mouse suture tissues (left panels), human suture cells (middle panels) and Kusa 4b 10 osteoblasts (right panels). Average expression stability values (M) of 12 candidate reference genes calculated by geNorm are shown (top row). A lower M value indicates a higher stability. An M value of <0.5 has been reported to be typical of stable genes expressed in relatively homogenous sample panels (2). Determination of the optimal number of reference genes for normalization was done using geNorm to calculate the pairwise variation value (V) between the normalization factors NF_n and NF_{n+1}, where NF_n is the geometric mean of the n most stable reference genes and NF_{n+1} the stepwise inclusion of the next most stable gene (bottom row). A V value below 0.15 is considered acceptable provided the inclusion of the next most stable gene does not cause a significant change in V (2).
