# Supplementary material for: Bone to pick: the importance of evaluating reference genes for RT-qPCR quantification of gene expression in craniosynostosis and bone-related tissues and cells
Source: BMC Res Notes. 2012 May 8;5:222. doi: 10.1186/1756-0500-5-222 (PMC3476976; doi:10.1186/1756-0500-5-222)
Supplement: Additional file 4 — Reference gene stability ranking in three bone-related experimental groups using Normfinder analysis. [file 1756-0500-5-222-S4.pdf]

Bone to pick: the importance of evaluating reference genes for RT-qPCR quantification of gene expression in craniosynostosis and bone-related tissues and cells

Xianxian Yang, Jodie T Hatfield, Susan J Hinze, Xiongzheng Mu, Peter J Anderson and Barry C Powell

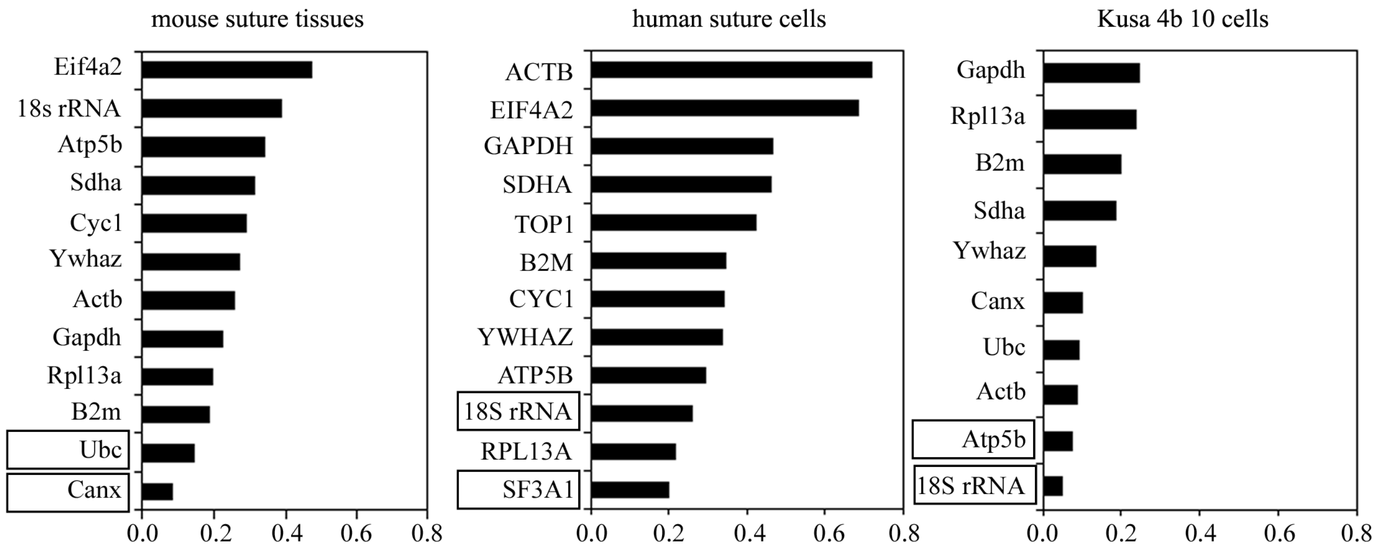

Additional file 4

Reference gene stability ranking in three bone-related experimental groups using Normfinder analysis

Normfinder analyses were carried out on craniosynostosis-related mouse suture tissues (left panel), human suture cells (middle panel) and Kusa 4b 10 osteoblasts (right panel). Average expression stability values of 12 candidate reference genes calculated are shown in graph format. A lower value indicates a higher stability. Each sample set was split into two groups to determine the inter- and intragroup variation in order to calculate the best two gene combination for normalization. The mouse suture tissues were split into wildtype vs mutant samples, the human cells were split into patent vs fusing or fused derived cells and the Kusa cells were divided into induced and uninduced samples. The final results are boxed above.
